# 5-Fluorouracil-induced RNA stress engages a TRAIL-DISC-dependent apoptosis axis facilitated by p53

**DOI:** 10.18632/oncotarget.6030

**Published:** 2015-10-24

**Authors:** Birce Akpinar, Ethiene V. Bracht, Dorin Reijnders, Barbora Safarikova, Iva Jelinkova, Alf Grandien, Alena Hyrslova Vaculova, Boris Zhivotovsky, Magnus Olsson

**Affiliations:** ^1^ Division of Toxicology, Institute of Environmental Medicine, Karolinska Institutet, Stockholm, Sweden; ^2^ Department of Cytokinetics, Institute of Biophysics, Academy of Sciences of the Czech Republic, v.v.i., Brno, Czech Republic; ^3^ Department of Medicine, Center for Infectious Medicine, Karolinska Institutet, Karolinska University Hospital–Huddinge, Stockholm, Sweden

**Keywords:** colon cancer, apoptosis, 5-fluorouracil, p53, necrosis

## Abstract

Despite recent advances in targeted therapeutics, administration of 5-fluorouracil (5-FU) remains a common clinical strategy for post-surgical treatment of solid tumors. Although it has been proposed that RNA metabolism is disturbed by 5-FU treatment, the key cytotoxic response is believed to be enzymatic inhibition of thymidylate synthase resulting in nucleotide pool disproportions. An operating p53 tumor suppressor signaling network is in many cases essential for the efficiency of chemotherapy, and malfunctions within this system remain a clinical obstacle. Since the fate of chemotherapy-insensitive tumor cells is rarely described, we performed a comparative analysis of 5-FU toxicity in p53-deficient cells and conclude that p53 acts as a facilitator rather than a gatekeeper of cell death. Although p53 can act as a regulator of several cellular stress responses, no rerouting of cell death mode was observed in absence of the tumor suppressor. Thus, the final death outcome of 5-FU-treated *p53^−/−^* cells is demonstrated to be caspase-dependent, but due to a slow pace, accumulation of mitochondrial reactive oxygen species contributes to necrotic characteristics. The oligomerization status of the p53 target gene DR5 is determined as a significant limiting factor for the initiation of caspase activity in an intracellular TRAIL-dependent manner. Using several experimental approaches, we further conclude that RNA- rather than DNA-related stress follows by caspase activation irrespectively of p53 status. A distinct 5-FU-induced stress mechanism is thereby functionally connected to a successive and discrete cell death signaling pathway. Finally, we provide evidence that silencing of PARP-1 function may be an approach to specifically target p53-deficient cells in 5-FU combinatorial treatment strategies. Together, our results disclose details of impaired cell death signaling engaged as a consequence of 5-FU chemotherapy. Obtained data will contribute to the comprehension of factors restraining 5-FU efficiency, and by excluding DNA as the main stress target in some cell types they propose alternatives to currently used and suggested synergistic treatment regimens.

## INTRODUCTION

Administration of 5-fluorouracil (5-FU, fluorouracil) is a common post-surgical treatment regimen used for several categories of solid tumors, and especially for patients suffering from colorectal cancers (CRC). The therapeutic potential of 5-FU was primarily described as an effect of its metabolic conversion into fluorodeoxyuridine monophosphate (FdUMP), a suicide substrate for thymidylate synthase (TS), and as such able to starve cells from deoxythymidine monophosphate (dTMP) [1]. By this means, a rate-limiting step obligatory for DNA replication and repair is eliminated and a link to cell death mechanisms provided. Fluorouridine triphosphate (FUTP), on the other hand, can be misincorporated into RNA. In fact, drug activity patterns derived from gene expression profiles in 60 human cancer cell lines clustered 5-FU to RNA synthesis inhibitors [2]. Misincorporation of 5-FU metabolites into RNA transcripts and genomic DNA appears to occur simultaneously in tumor cells, but no significant correlation between the magnitude of integration into either species and the response to 5-FU therapy has yet been found [3]. Hence, despite the extensive clinical use of 5-FU, the relative importance of each stress target is not clearly established.

Similar to other chemotherapeutic agents, 5-FU induces cell cycle arrest and apoptosis in sensitive tumor cell lines. While an arrest in the G1/S cell cycle phase boundary was reported to occur as a result of checkpoint kinase 1 (Chk1)-mediated Cdc25A inactivation [4], the mechanisms leading to the commencement of apoptosis signaling are poorly understood. Since the p53 tumor suppressor plays a central role in DNA damage responses, including DNA repair, the cell cycle regulations and apoptosis, a key focus has been on how these processes relate to 5-FU toxicity. For instance, gene expression analyses identified mitochondrial ferredoxin reductase as a p53 target gene inducing cell death via mitochondrial reactive oxygen species (ROS) formation [5]. *In vivo* and *in vitro* studies also suggest that 5-FU-treated cancer cells conform to a p53-dependent extrinsic apoptosis mechanism directed by receptors included in the tumor necrosis factor family (TNF) [6, 7]. Yet, although p53 status was proposed as an accurate indicator of CRC prognosis and 5-FU therapy response *in vivo* and *in vitro* [8–10], it is still a matter of debate. For example, a correlation between mutations in the conserved p53 DNA binding region and treatment efficacy indicated that this aspect of protein function is not a clinically useful predictive marker for the response of Dukes’ C stage colon cancers to 5-FU chemotherapy [11]. Nevertheless, in experimental models where p53 status has been used to explain gross differences in 5-FU responses, it is evidently clear that cells harboring p53-insufficiency are also affected by treatment [9, 12]. In contrast to the analysis of functional stress pathways where the silencing of key regulatory elements mostly serves as controls, we have explored in detail the kinetics and underlying mechanisms of p53-independent cell death by using parental and genetically-modified HCT116 cells, one of the most common *in vitro* systems for 5-FU toxicity analyses. By this experimental approach, we clarified the role of the tumor suppressor in several aspects of drug toxicity, ranging from initial stress target point to molecular mechanisms of apoptosis and cell fate. We also provide evidences supporting a mechanism by which tumor cells lacking p53 are sensitized to 5-FU combinatorial treatment strategies targeting PARP-1.

## RESULTS

### p53 facilitates the appearance of apoptotic markers in 5-FU-treated HCT116 cells

**Figure 1:**
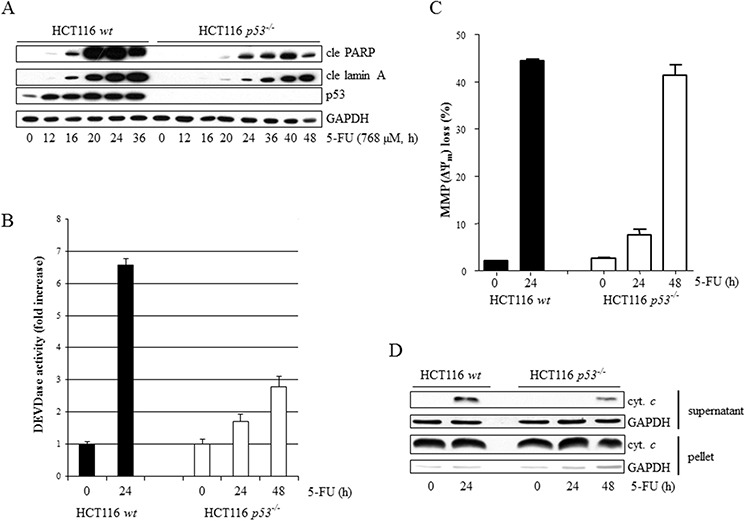

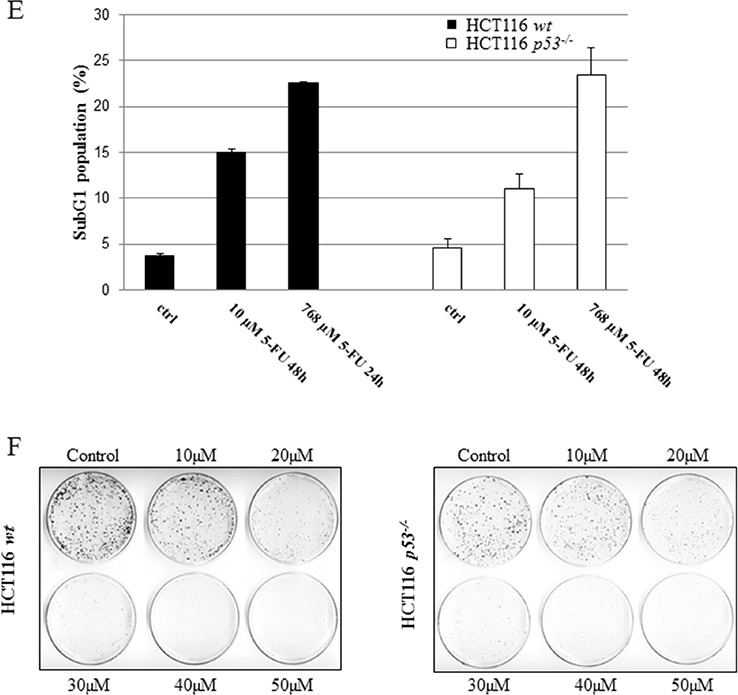
Comparative analysis of 5-FU-induced apoptosis in wt and p53-deficient HCT116 cells The apoptotic indicators, cleaved PARP and lamin A (cle PARP, cle lamin A), were analyzed at multiple time points ranging from 0 to 36 h post-treatment initiation in *p53*-proficient and 0 to 48 h in *p53*-deficient HCT116 cells using immunoblotting of SDS-PAGE-separated cell lysates **A.** Caspase-3/-7-like activities were measured by monitoring the liberation of AMC from the synthetic fluoro-conjugated peptide substrate motif DEVD (DEVD-AMC) **B.** Drug effects with respect to loss of mitochondrial membrane potential (ΔΨ_m_) in live cells were examined by FACS analysis of mitochondrial TMRE accumulation **C.** HCT116 *wt* and *p53^−/−^* cells, treated with 768 μM 5-FU for time periods indicated, were harvested and subjected to separation into cytoplasmic and nuclear/mitochondrial fractions, enabling immunoblotting of released cytochrome *c* **D.** Cells, treated with 5-FU (10 μM, 48 h or 768 μM, 24 h), were fixed in 70% ethanol and stained with propidium iodide before FACS determination of sub G1 populations **E.** Clonal survival of eighteen HCT116 *wt* or *p53^−/−^* cells/cm^2^ in response to 5-FU treatment (48 h), ranging from 10 to 50 μM **F.** GAPDH served as a marker for equal sample loading in (A) and (D), and also as an indicator of fractionation efficacy in (D).

HCT116 has been verified as type II cells [13], stating that mitochondrial destabilization is required for efficient apoptosis. The HCT116 parental (*wt*) cell line and its derivate lacking p53 (HCT116 *p53^−/−^*) were, therefore, treated with 5-FU and analyzed with respect to loss of mitochondrial membrane potential (MMP, ΔΨ_m_), release of cytochrome *c* into the cytosol, DEVDase (caspase-3/-7-like) activity and poly(ADP-ribose) polymerase-1 (PARP-1) cleavage. Notably, although all markers appeared earlier and were more pronounced in *wt* cells, they could also be readily detected independently of p53 function (Figure 1A–1D). Interestingly, although the DEVDase activity in HCT116 *p53^−/−^* cells at 48 h of treatment only reached approximately half the intensity compared to their *wt* counterpart at 24 h (Figure 1B), similar rates of overall cell death were quantified by FACS analysis of the subG1-population in both data sets (Figure 1E). Thus, the consequence of p53 deficiency in this context is indeed a suboptimal apoptotic signaling cascade which, however, generates substantial cell death in a timely delayed manner. Apart from experimental conditions where efficient cell death was assured by using high doses of 5-FU (768 μM), treatment for 48 h using lower concentrations of the drug (10 μM) also generated substantial apoptosis, both in the absence or presence of p53 (Figure 1E). Similar colony formation capacity in both cell types over a range of 5-FU concentrations was observed (Figure 1F). However, the presence of a pan-caspase inhibitor (zVAD-fmk) does not rescue tumor cell colony formation in p53-deficient cells (Supplementary figure 1A). This indicates that during culture conditions where cells are seeded sparse (<20 cells/cm^2^) 5-FU treatment does not engage cell death pathways, as is the case in “normal culture settings” (5 × 10^4^ cells/cm^2^), but primarily follows by a p53-independent cell cycle arrest. In support of this observation, cells were arrested in a G1/S cell cycle boundary within 8 hours of treatment irrespectively of p53 (Supplementary figure 1B).

### Despite necrotic characteristics 5-FU-treated HCT116 p53^−/−^ cells primarily die by caspase-dependent apoptosis

**Figure 2:**
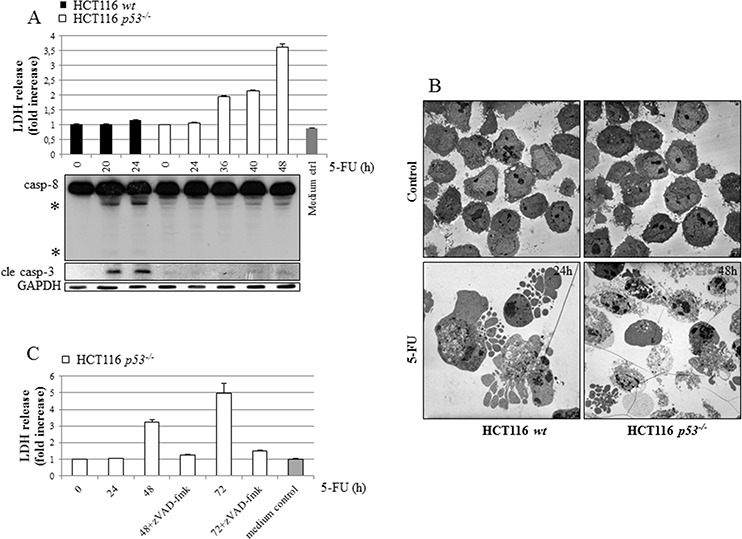

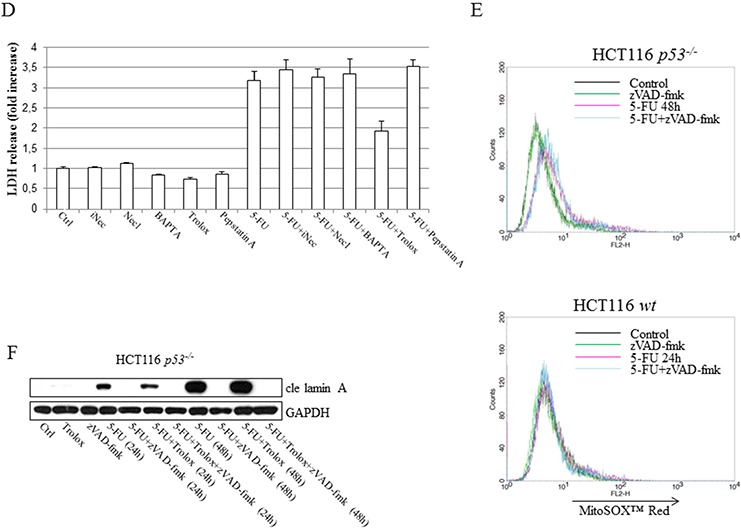
Irrespectively of p53 status, HCT116 cells die by apoptosis but characteristics of necrosis develop in the absence of the tumor suppressor as a consequence of ROS formation HCT116 cells with different p53 status were treated with 5-FU (768 μM), and at time points indicated cellular LDH release and SDS-PAGE-mediated detection of active subunits relating to caspases-3 (cle casp-3) and -8 were performed in parallel **A.** Representative transmission electron microscope images of sections prepared from control and 5-FU-treated *wt* and *p53^−/−^* cells. An isolation of dead cells was performed in treated samples by collecting floating cells prior to fixation and section preparation **B.** Analysis of LDH-release from HCT116 *p53^−/−^* cells at 24, 48 and 72 h post-5-FU treatment (768 μM), either in the presence or absence of the pan-caspase inhibitor zVAD-fmk (10 μM) **C.** In **D.** HCT116 *p53^−/−^* cells were treated with 5-FU for 48 h, either alone or in the presence of chemical inhibitors of necrosis, inactive NEC (iNEC, 100 μM), NEC (100 μM), BAPTA (10 μM, last 24 h only), Trolox (50 μM) or pepstatin A (7 μM), in advance of LDH-release analysis. Mitochondrial ROS was measured by FACS analysis of control and 5-FU-treated MitoSOX™ Red stained HCT116 *wt* (24 h) and *p53^−/−^* (48 h) cells, cultured either in the presence or absence of zVAD-fmk **E.** Lysates isolated from cells treated with 5-FU, either alone or in combination with 50 μM Trolox, were subjected to SDS-PAGE in order to investigate whether or not ROS reinforces apoptosis. Detection of cle lamin A served as an marker for apoptosis **F.** GAPDH probing indicates equal sample loading in (A) and (F). In (A), processed caspase-8 fragments are indicated with asterisks.

Previously, we identified a novel signaling route occurring as a consequence of 5-FU-induced entry of extracellular Ca^2+^, where calmodulin served as a link to p53 phospho-activation and apoptosis [6]. Ca^2+^-overload may also induce programmed necrosis resulting from a calpain-dependent mitochondria-nuclei translocation of the apoptosis-inducing factor (AIF) [14]. We, therefore, speculated that in p53-deficient HCT116 cells where apoptosis is suboptimal, alternative cell death modes may become more pronounced. Indeed, 5-FU-treated HCT116 *p53^−/−^* cells display several molecular and biochemical characteristics of necrosis, including plasma membrane disintegration, determined by extracellular lactate dehydrogenase (LDH) and transmission electron microscopy (TEM) (Figure 2A and 2B). These features were in sharp contrast to treated HCT116 *wt* cells where plasma membranes remained intact in apoptotic cells (Figure 2A and 2B). Importantly, in absence of p53 cellular release of LDH was completely abrogated by the pan-caspase inhibitor zVAD-fmk, suggesting that the predominant cell death mode is indeed caspase-dependent (Figure 2C). To clarify how necrotic signatures evolve in apoptotic HCT116 *p53^−/−^* cells, 5-FU combinatorial treatments using chemical inhibitors to potential necrotic conduits, including the Ca^2+^ chelator BAPTA (1,2-bis (o-aminophenoxy)ethane-N,N,N’,N’-tetraacetic acid), the RIP1-kinase inhibitor necrostatin 1 (NEC1), the antioxidant Trolox, and the inhibitor of lysosomal acid proteases pepstatin A were performed. As Trolox was the only compound able to limit LDH release, we concluded that ROS is a mediator of necrotic characteristics in 5-FU-treated cells harboring a disintegrated apoptotic program (Figure 2D). It is important to note that zVAD-fmk in the absence of p53 by no means obstructs the generation of mitochondrial ROS, nor does Trolox inhibit 5-FU-induced apoptosis, as determined by FACS analysis of MitoSOX™ Red-stained cells and SDS-PAGE, respectively (Figure 2E and 2F). Mitochondrial release of AIF could not be detected in organelle fractions isolated from treated cells, thus excluding this as being an important factor for the necrotic features described (Supplementary figure 2). Moreover, necrotic morphology determined by TEM and LDH-release appears simultaneously as the initiation of cell death and the activation of caspases-8 and -3 (Figure 2A, Supplementary figure 3). In the presence of p53, an efficient apoptotic program does not allow ROS formation (Figure 2E). The distinct necrotic features of treated HCT116 *p53^−/−^* cells, on the other hand, may be an indirect consequence of suboptimal apoptosis and mediated by mitochondrial ROS, elevated in cells by mechanisms uncoupled from the apoptotic program.

### DR5-DISC determines apoptosis efficiency in a p53- and TRAIL-dependent manner

**Figure 3:**
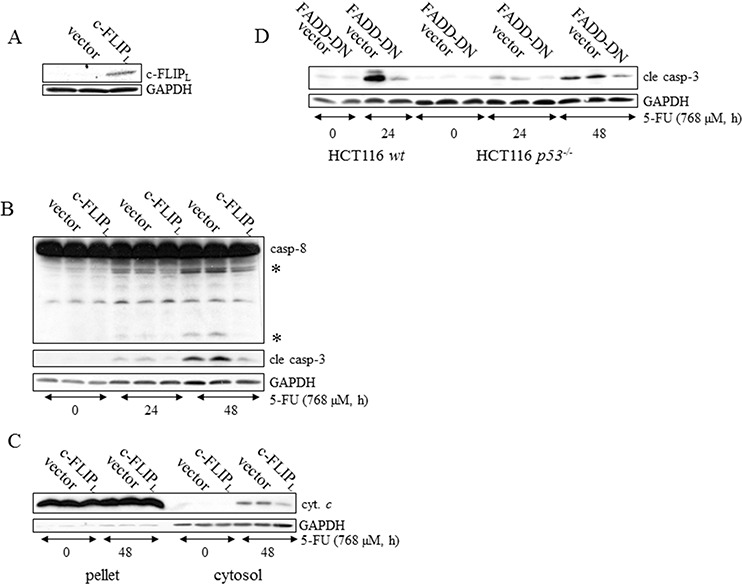
DISC activation is a prerequisite for caspase-dependent apoptotic signaling in p53-deficient HCT116 cells c-FLIP_L_, FADD-DN or empty expression vectors were stably introduced into *wt* and *p53^−/−^* HCT116 cells by retroviral transduction. SDS-PAGE- analysis of c-FLIP_L_ in lysates harvested from untreated HCT116 *p53^−/−^* cells, transduced with empty or c-FLIP_L_ expression vectors **A.** Non-transduced, empty vector-containing and c-FLIP_L_ expressing HCT116 *p53^−/−^* cells were harvested at the indicated time after 5-FU treatment and immunoblotting served to detect the processing of apical caspase-8 and effector caspase-3 in **B.** Alternatively, cells were subjected to fractionation into cytoplasmic and mitochondrial/nuclei protein pools for analysis of cytochrome *c* release **C.** Control, empty vector-containing and FADD-DN expressing HCT116 *p53^−/−^* cells and HCT116 *wt* cells harboring empty and FADD-DN expression vectors were incubated in the presence of 768 μM 5-FU. After isolation of total protein lysates, immunoblot analysis, using a specific antibody directed to the active p18 caspase-3 fragment, served as an indicator of apoptosis **D.** Probing of GAPDH was used to confirm equal loading of the samples (A-D) or as an indicator of fractionation efficacy (C). In (B), processed caspase-8 fragments are indicated with asterisks.

**Figure 4:**
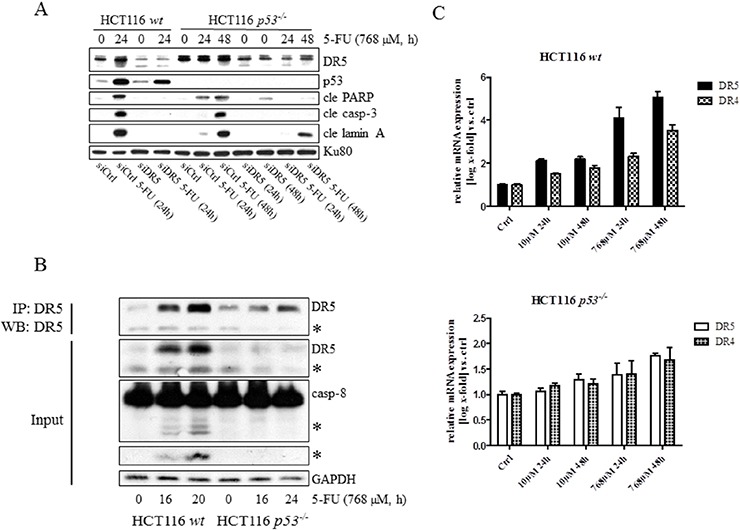

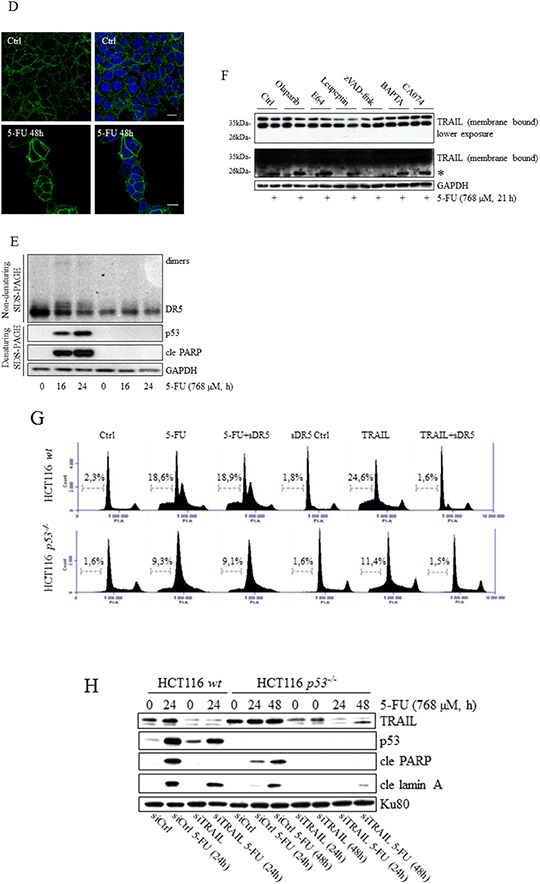
Independently from p53, plasma membrane accumulation of DR5 occurs in response to 5-FU treatment, and silencing of the protein reduces drug-induced apoptosis The effect of 5-FU with respect to the processing of caspase-3, PARP and lamin A in HCT116 *wt and p53^−/−^* cells was analyzed by immunoblotting, using total protein lysates from cells in which DR5 had been silenced by means of RNAi (30 nmol) **A.** Western blot analysis of lysate input and DR5 IP generated from control and 5-FU-induced HCT116 *p53^−/−^* and *wt* cells. DR5 and caspase-8 processing is shown in input samples **B.** cDNA from control and treated (10 and 768 μM 5-FU) HCT116 *wt* and *p53^−/−^* cells were generated and mRNA expression analyses with respect to DR4 and DR5 were accomplished by RT-PCR using specific primers **C.** Along with control cells, HCT116 *p53^−/−^* cells induced with 5-FU for 48 h were fixed in 4% formaldehyde and stained with a DR5-specific antibody (green). Cell nuclei were counterstained by DAPI (blue) and samples analyzed using confocal microscopy **D.** Western blot analysis of DR5-dimerization using non-reduced protein lysates isolated from control and 5-FU-treated HCT116 *wt* and *p53^−/−^* cells. Reduced samples were separated in SDS-PAGE for detection of p53 and cle PARP **E.** Appearance of processed TRAIL was investigated by SDS-PAGE using HCT116 *wt* cell samples, either treated with 5-FU alone or in combination with olaparib (500 nM), E64 (10 μM), leupeptin (100 μM), zVAD-fmk (10 μM), BAPTA (10 μM) or CA074 (10 μM) **F.** Cells, treated either with 5-FU (30 μM, 48 h) or recombinant TRAIL (10 ng/ml) alone, or in combination with a soluble recombinant DR5 (2 μg/ml; sDR5), were fixed in ethanol and stained with propidium iodide before FACS determination of subG1 populations **G.** Cells were transfected with siRNA targeting TRAIL (30 nmol) 24 h in advance of 5-FU-treatment. Protein lysates were isolated and separated by SDS-PAGE in order to analyze TRAIL, cle lamin A and p53 using specific antibodies **H.** GAPDH and Ku80 were used as a control for equal loading of samples in (A, B, E, F and H). Processed TRAIL and caspase-8 as well as the short isoforms of DR5 are indicated with asterisks (B and F). Bars, 10 μm (D).

Chemotherapeutic agents, such as platinum compounds and doxorubicin, have been reported to upregulate the TNF family death receptors DR4 and DR5 due to p53 transcriptional activity. As a result, chemotherapy in combination with TRAIL frequently augments cell death rates of cancer cells [15, 16]. Since apoptosis in response to 5-FU was previously shown to depend on the TNF-family receptors *in vivo* and *in vitro*, as well as in the absence of TRAIL co-treatment, we examined whether or not p53 is critical for DISC-mediated cell death. In support of this assumption a prominent apical caspase-8 processing was detected in HCT116 *p53^−/−^* cells (Supplementary figure 4A). Using a RNAi approach, we were also able to conclude that caspase-8 is an upstream and essential factor for effector caspase activation in these cells (Supplementary figure 4B). To verify that caspase-8-activation occurs at the level of DISC formation, we investigated apoptotic markers appearing in response to 5-FU in HCT116 *p53^−/−^* cells stably overexpressing either the regulatory cellular caspase-8 (FLICE)-like inhibitory protein (c-FLIP_L_) or a truncated version (dominant negative) of the Fas-associated death domain (FADD) adaptor protein (FADD-DN). Since both of these factors harbor a capacity to obstruct correct DISC formation, reduced levels of processed caspases and free cytosolic cytochrome *c* in the case of ectopic c-FLIP_L_ expression, and a decrease in active caspase-3 in the case of FADD-DN overexpression, justify our conclusion that 5-FU also induces DISC-mediated cell death in the absence of p53 (Figure 3A–3D). Previously, we revealed that DR5, but not the Fas receptor, is fundamental for effective apoptosis in HCT116 *wt* cells [6]. Similar to their *wt* counterpart, 5-FU-induced apoptosis was abrogated by RNAi-silencing of DR5 also in HCT116 cells lacking p53, concluding that the absence of the tumor suppressor does not force cells to alter the death signaling pathway (Figure 4A). By using RNAi methodology, we also concluded DR4 as important for 5-FU-induced apoptosis, irrespectively of p53 function (Supplementary figure 5). Consistent with current data identifying DR4 and DR5 as p53 transcriptional target genes [17, 18], enhanced mRNA levels and accumulation of the corresponding proteins were more prominent in HCT116 *wt* cells after 24 h 5-FU treatment (Figure 4B and 4C, Supplementary figure 5). Interestingly, immunoblotting of cell lysates and immunoprecipitations (IP) using DR-specific antibodies also indicated a minor increase of the receptors independently of p53. These data were supported by an equivalent enhancement of the DR4 and DR5 mRNA's and also by the localization of the DR5 to plasma membranes in treated cells (Figure 4B–4D). Thus, DR4 and DR5 expression may be adjusted by both p53-dependent and -independent mechanisms where the latter may be a consequence of ER stress (Supplementary figure 6) [19]. To verify that DR5 is indeed oligomerized in response to treatment, protein lysates were isolated under non-reducing conditions and DR5 dimer formation analyzed by SDS-PAGE (Figure 4E). Since the presence of p53 supported dimer establishment, DR5 activity is established as one of the most upstream limiting apoptotic factor in the cell system analyzed. However, although the importance of DR4 and DR5 for 5-FU-induced apoptosis is clearly emphasized by these results, process(es) separated from the upregulations of DR4 and DR5 *per se* may contribute to activation of the receptors. In the current literature, TRAIL is postulated as the sole DR4/5-activating ligand. Apart from membrane-bound TRAIL, specific antibodies used in the study also detected a ~24 kDa protein fragment in high exposed immunoblotting, which is similar to the reported molecular weight of soluble TRAIL [20]. As this particular protein fragment appeared only in samples isolated from 5-FU-treated cells, we reasoned that there might be a connection to DR4/5 stimulation. Yet, among chemical inhibitors with the potential to delimit or enhance proteolytic cleavage, only zVAD-fmk blocked the generation of the fragment, leading to the conclusion that its appearance is a consequence of caspase activity (Figure 4F). Importantly, recombinant DR5 lacking the integral membrane domain, thus acting as a soluble inhibitor of extracellular TRAIL, failed to interfere with the cell death progression in 5-FU-treated cells, while blocking TRAIL-stimulated apoptosis completely, as shown by FACS analysis of Sub G1-populations (Figure 4G), and apoptotic markers in SDS-PAGE (Supplementary figure 7). In contrast, RNAi targeting of TRAIL abrogated cell death irrespectively of p53 function (Figure 4H). Hence, although mechanisms for DR4 and DR5 activation supported by intracellular, membrane bound TRAIL are lacking in the literature, our results demonstrate their existence. In conclusion, these experiments indicate 5-FU as a chemotherapeutic drug enabling apoptosis by p53-facilitated DISC oligomerization in a TRAIL-dependent manner.

### 5-FU induces apoptosis through an RNA-stress-related pathway

**Figure 5:**
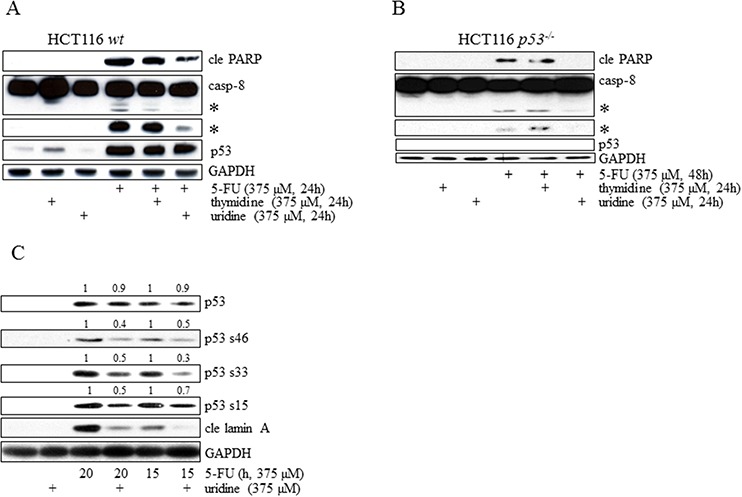
Irrespectively of p53 status, induction of apoptosis in HCT116 cells occurs as a consequence of 5-FU-mediated RNA-stress HCT116 *wt* **A.** and *p53^−/−^* **B.** cells were treated with 5-FU alone for 24 and 48 h, respectively, in the absence or in the presence of either thymidine or uridine. Total cell lysates were then separated by SDS-PAGE and analyzed by immunoblotting with respect to p53, caspase-8 and cle PARP. In a similar experimental set-up, phospho-activation of p53 (S15 S33 and S46) was analyzed by SDS-PAGE in HCT116 *wt* cells treated with 5-FU (375 μM) alone (15 or 20 h) and compared to the p53 phospho-status in lysates isolated from cells co-treated with an equimolar concentration of uridine. Quantifications of p53 and its phospho-variants were accomplished through normalization with GAPDH. 15 and 20 h treatments are normalized separately **C.** Probing of GAPDH was used to confirm equal loading of the samples (A–C). Processed caspase-8 is indicated with asterisks (A and B).

In contrast to other chemotherapeutic agents, metabolized 5-FU elicits not only damage to DNA but also influences RNA metabolism. Discrimination between these stress targets can be accomplished by analysis of whether thymidine or uridine reverses 5-FU-induced toxicity in cells [9, 21]. 5-FU desensitization of cells by thymidine can be explained by enhanced dTMP production through the thymidine kinase (TK) salvage pathway, thereby reducing the requirement for TS activity. Adding an excess of uridine to the system, on the other hand, reduces RNA-misincorporation of FUTP. Our results confirm data indicating HCT116 cells as being primarily susceptible to 5-FU-induced RNA-related stress and consequently toxicity-liberated by the addition of equimolar concentrations of uridine to the system (Figure 5A) [9]. By using the same approach we also concluded that in the absence of p53 the primary 5-FU stress target point remained unaltered (Figure 5B). Uridine also inhibits 5-FU-induced LDH-release in HCT116 *p53^−/−^* cells, thus validating our data concluding that slow apoptosis proceedings may result in necrotic cell features (Supplementary figure 8). Together these data clearly indicate a p53-facilitated functional link between RNA lesions and DISC-regulated apoptosis. They also support the idea of p53 as mainly functioning in the regulation of apoptotic mechanisms and not being involved in processes dictating the relative RNA/DNA-targeting of 5-FU metabolites. It is clear, however, that depending on their origin, tumor cell types respond differentially to 5-FU metabolites [21]. Intriguingly, levels of the p53 protein remain in 5-FU-uridine co-treatment (Figure 5A and 5C). Phospho-activation of p53 S15, S46 and S33, on the other hand, was reduced compared to cells treated with 5-FU alone (Figure 5C), thus verifying that uridine is acting upstream of p53.

**Figure 6:**
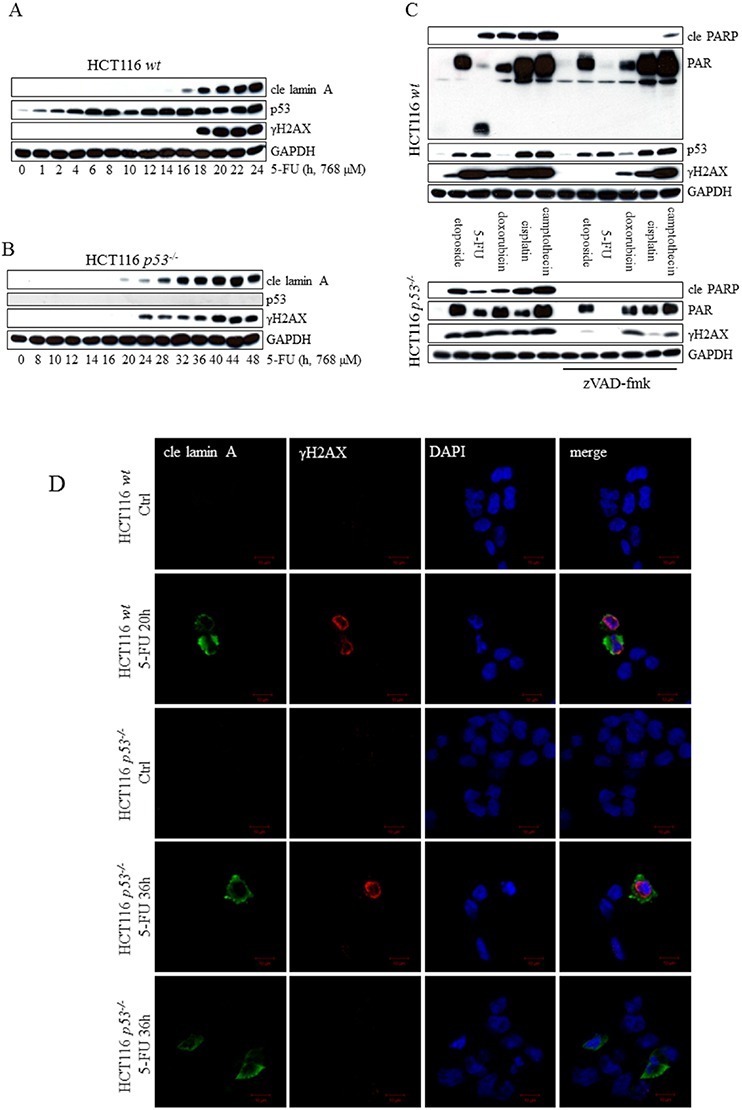
Markers of DNA damage appear as a consequence of 5-FU-induced caspase-activity Analysis of p53, cle lamin A and γH2AX in SDS-PAGE separated protein lysates from HCT116 *wt* **A.** and HCT116 *p53^−/−^* cells **B.** harvested at multiple time points post-5-FU-treatment. HCT116 *wt* and *p53^−/−^* cells were treated for 24 and 48 h, respectively, with the chemotherapeutic agents indicated, either alone, or in the presence of 10 μM zVAD-fmk. Total cell lysates were then analyzed by immunoblotting with respect to the apoptotic marker cle PARP and the DNA-damage indicators γH2AX and PAR **C.** Probing of GAPDH was used to confirm equal loading of the samples (A–C). Along with control cells, HCT116 cells (*wt* and *p53^−/−^*) induced with 5-FU for 20 and 36 h, respectively, were fixed in 4% formaldehyde immunostained using cle lamin A (green) and γH2AX (red) specific antibodies. Cell nuclei were counterstained by DAPI (blue) and samples analyzed by confocal microscopy **D.** Bars, 10 μm.

We reasoned that if a cell line subjected to 5-FU suffers from the inhibition of TS, it is likely that imbalances of the nucleotide pool would lead to DNA double-strand breaks (DSB) through perturbations in the replication and repair mechanisms. Massive phospho-activation of the histone H2AX (γH2AX), one of the widely used markers for DSB, were indeed detected in HCT116 *wt* and p53-deficient cells but seem to appear simultaneously with lamin cleavage (Figure 6A and 6B). Therefore, we next investigated whether DNA damage is a cause or consequence of 5-FU-induced apoptosis. In the presence or absence of a functional p53, the addition of zVAD-fmk in combination with 5-FU reduced γH2AX to background levels, demonstrating DNA damage to be a secondary event occurring as a result of apoptosis (Figure 6C). In agreement with these results, immunostaining associated γH2AX with cleaved lamin A and condensed nuclei in treated cells (Figure 6D). In contrast, treatment with other chemotherapeutic agents, including doxorubicin, cisplatin and camptothecin, generated γH2AX activity, which remained in the presence of zVAD-fmk (Figure 6C). A similar pattern was also detected in etoposide (VP16)-treated HCT116 *p53^−/−^* cells. However, neither the apoptotic marker cle PARP nor γH2AX appeared in *wt* samples harvested 24 h post-etoposide induction. DNA stress results in the activation of PARP-1 and PARP-2, which, among other things, catalyzes the assembly of poly(ADP-ribose) (PAR) chains onto itself and adjacent nuclear proteins, a modification that serves to facilitate repair by attracting PAR-binding DNA repair factors [22]. By using PAR polymers as a marker for DNA lesions, our conclusion that DNA damage is a secondary event to 5-FU-induced apoptosis could be verified (Figure 6C). Similar to γH2AX, PAR remained in the presence of zVAD-fmk when cells were treated by the alternative chemotherapeutic agents. Finally, DNA-damage and apoptosis originating from 5-FU-mediated TS inhibition would theoretically be augmented by leucovorin (folinic acid), which is acting in synergy with 5-FU with respect to TS silencing. However, any enhancement of apoptosis was not detected by combinatorial treatment using leucovorin and 5-FU compared to 5-FU alone (Supplementary figure 9). In conclusion, these results indicate that irrespectively of p53 function and in contrast to a panel of other anti-tumor agents, primary DNA damage may not be the origin of 5-FU-induced apoptosis in some cell lines.

### Sensitization of tumor cell lines to 5-FU by chemical- and RNAi-mediated inhibition of PARP occurs specifically in the absence of p53

**Figure 7:**
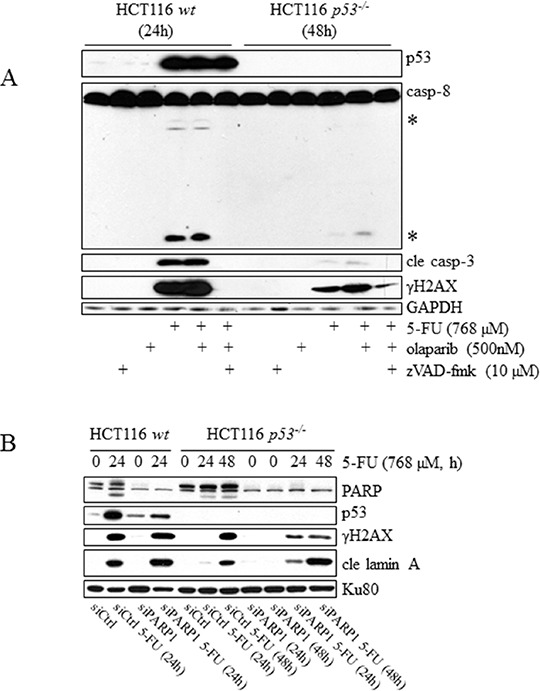

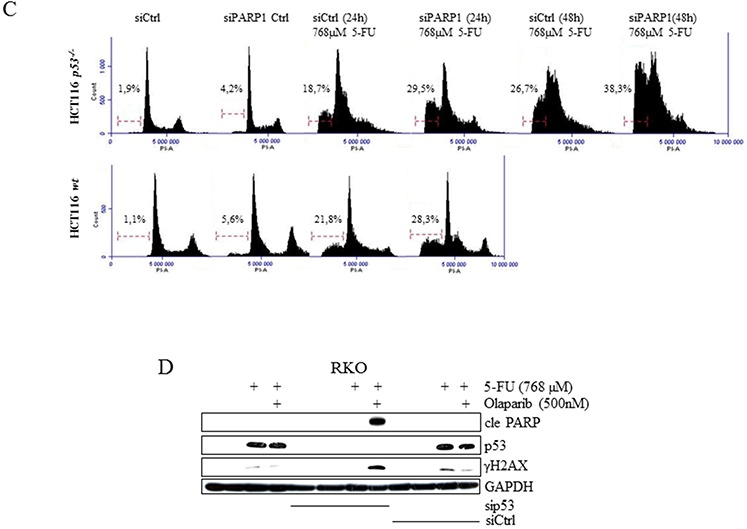
Sensitization of tumor cell lines to 5-FU by chemical and RNAi-mediated inhibition of PARP occurs specifically in the absence of p53 HCT116 *wt* and *p53^−/−^* cells, incubated in the presence of 5-FU alone for 24 and 48 h, respectively, or co-treated with 500 nM of the PARP inhibitor olaparib. Total cell lysates were then analyzed by immunoblotting with respect to p53, processing of caspases-8 and -3, and γH2AX **A.** The effect of 5-FU-treatment with respect to p53 and the processing of lamin A in HCT116 *wt* and *p53^−/−^* cells was analyzed by immunoblotting using total protein lysates from cells in which PARP1 had been silenced by means of RNAi (30 nmol). A non-targeting control siRNA was used to eliminate the possibility that transfections per se interfered with analysis outcome **B.** The consequence of 5-FU treatment with respect to sub G1 populations in HCT116 *wt* and *p53^−/−^* cells was analyzed by FACS using cells in which PARP 1 had been silenced by means of RNAi (30 nmol) **C.** Immunoblot-detection of DNA-damaging (γH2AX) and apoptotic (cle PARP) markers in 5-FU-treated RKO control cells, and in cells where p53 suppression was accomplished by means of RNAi. Comparisons of control, si control (sictrl) and sip53 transfected cells are outlined in **D.** GAPDH and Ku80 were used as a control for equal loading of samples (A, B and D). Processed caspase-8 is indicated with asterisks (A).

Several members of the PARP protein family, including PARP1-4 as well as tankyrase-1 and -2 have poly(ADP-ribosyl)ation activity, enabling transfer of ADP-ribose molecules to substrate proteins (PARylated proteins). This posttranslational modification is associated with several biological processes but its implication in the recognition and repair of DNA damage has so far been the main focus for future tumor therapies. In particular, inhibition of PARP-1 using small molecule inhibitors has received interest due to promising effects, both as a single agent and in combinatorial treatment regimens [23]. Although the marker for DNA DSB γH2AX is activated as a consequence of apoptosis in the current experimental system, studies using radiolabeled 5-FU have concluded that drug metabolites are misincorporated into DNA in a general fashion [24, 25]. At present, no such examination has been performed using HCT116 cells, but since uridine and not thymidine reverses 5-FU-induced apoptosis, any toxicity evolving from DNA stress might be suppressed by a shielding DNA repair capacity, resulting in cell death as a consequence of other stress targets such as RNA. Accordingly, manipulation of DNA repair systems could be a feasible approach to enhance the apoptotic 5-FU response in some tumor cells [26]. As PARP-1 has been implicated in several modes of single- and double-strand DNA repair, including base excision repair (BER), homologous recombination (HR) and non-homologous end joining (NHEJ) [23], we examined the combinatorial treatment effects of 5-FU and olaparib (PARP1–4 inhibitor) [27]. To our surprise, in comparison to 5-FU alone, an enhancement of cleaved caspase-3 and the processing of caspase-8 were detected by SDS-PAGE specifically in HCT116 *p53^−/−^* cells. Moreover, γH2AX signals also remained after the addition of zVAD-fmk to combinatory-treated cells, indicating primary DNA damage only in the absence of p53 (Figure 7A). With respect to apoptosis, a similar response pattern, using lamin A cleavage as an indicator of apoptosis and sub G1-populations of overall cell death, was confirmed by RNAi-targeting of PARP1, thereby identifying this particular member of the protein family as the main contributor of the effect (Figure 7B and 7C). Even if olaparib and siRNA contributed to 5-FU-induced apoptosis in HCT116 *p53^−/−^* cells, the overall response was still low compared to the *wt* counterpart treated with 5-FU as a single agent (Figure 7A–7C). However, using extended incubation times with lower 5-FU concentrations, we clearly demonstrated that the apoptotic ratio between p53-deficient cells treated with the olaparib combinatorial setting and 5-FU alone not only persisted but actually increased significantly, while it remained constant in *p53 wt* cells (Supplementary figure 10). In addition, to verify the general validity of our data, p53 was suppressed by siRNA in RKO cells, which were subsequently treated with either 5-FU or olaparib alone, or their combination. Similar to what was concluded in HCT116 cells, a synergetic effect of the drugs was detected only in RKO cells transfected with p53 siRNA (Figure 7D). Although a more detailed mechanistic explanation for this finding remains to be established, the data may certainly be of importance for the design of future combinatorial tumor therapies.

## DISCUSSION

The metabolic activation of 5-FU potentiates several distinct stress conditions within cancer cells. As TK misrecognizes FdUMP as a substrate, its enzymatic silencing renders dTTP starvation and elevated dUTP pools, which in turn lead to nucleotide misincorporation and mutagenesis, as well as to defects in DNA replication and repair processes. In addition, several lines of evidences support a model where drug toxicity is generated from RNA-related effects. While early works associated tumor cell lethality with the incorporation of 5-FU into RNA [28, 29], recent achievements have further specified mechanisms leading to malfunctioning rRNA processing and pre-mRNA splicing defects as explanations for drug-induced toxicity [30, 31]. Although experimental evidences are lacking, the fast post-transcriptional reduction of ribosomal proteins and translational capacity in treated cells might reflect early rRNA processing disorders [32]. Apart from apoptosis, stress conditions resulting from 5-FU treatment include the unfolded protein response (UPR) succeeding endoplasmic reticulum (ER) stress and stimulation of autophagy [33, 34]. To our knowledge, a more complete view of how these response patterns interrelate has not yet been presented. Interestingly, however, as a way to maintain cell homeostasis, ER stress may reduce translational capacity via the inhibition of rRNA synthesis and stimulation of autophagy [35], processes which are associated with 5-FU treatment.

**Figure 8:**
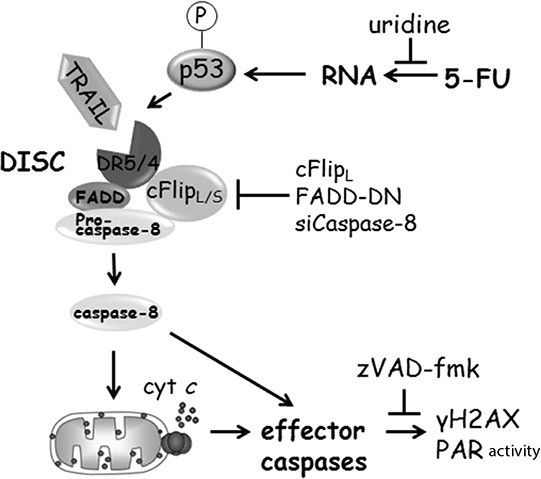
Schematic illustration of 5-FU-induced apoptotic signaling in HCT116 cells (for details, see text)

Our aim was to establish a functional connection leading from a specific drug target point to a discrete cell death mechanism and further describe how p53-deficiency interferes with this process (Figure 8). Obtained data supports the notion that apoptotic mechanisms induced by 5-FU originate from RNA-related stress, at least in selected cell types. In view of the fact that uridine but not thymidine liberates cells from apoptotic stress stimulated by 5-FU-metabolites in a p53-independent manner, the tumor suppressor status of cells may not be an answer to why different cell lines seem to differ with respect to drug stress origin. Results from the functional assay using free nucleosides were confirmed by the disclosure of γH2AX- and PAR-appearances as secondary to apoptotic initiation. Phospho-activation of H2AX occurring as a consequence of apoptosis has been investigated in some detail by others. In line with our findings, it has been reported that DISC-mediated apoptosis may stimulate not only γH2AX but also other factors associated with the DNA damage response [36]. The expression pattern and the activating kinase were shown to diverge between apoptosis- and DNA damage-stimulated phosphorylation of histone H2AX. Importantly, in contrast to apoptotic caspase-dependent Chk2-activity, γH2AX did not amplify the proceedings of cell death [36, 37], which is in line with our previous observations [6]. Instead, apoptotic H2AX activity seems to be important for nuclear morphological changes, and especially caspase-activated DNase (CAD)-mediated DNA-ladder formation [37]. To what extent PAR also participates in any such processes is currently not known. In contrast to 5-FU-treated cells, where both PAR and γH2AX were reduced below detection level in the presence of a pan-caspase-inhibitor, signals decreased but remained measurable in experimental conditions embracing co-treatment with alternative chemotherapeutic agents. Apart from serving as positive controls for primary DNA damage, diminished γH2AX in these samples also indicate that apoptosis- and DNA-damage-stimulated phosphorylation of the histone may occur simultaneously in cells. PAR, on the other hand, persisted when zVAD-fmk was combined with all drugs except 5-FU, thus implying caspase-dependent PAR formation as being specific to this particular treatment regimen. The significance of the DISC for 5-FU-induced apoptosis was accomplished by no less than five discrete approaches: FLIP_L_ and FADD-DN overexpression, as well as RNAi-targeting of DR4, DR5 and caspase-8. Combined with the fact that uridine hindered caspase-8 processing and that zVAD-fmk reduced markers of DNA damage to background levels, the current data set is indicating a link between 5-FU-induced RNA-stress and the apoptotic initiating point constituted by DR4 and DR5 (Figure 8). How, then, is death receptors oligomerized? The conclusion from our data set is that the cognate receptor ligand TRAIL is obligatory for DISC aggregation and initiation of the caspase cascade. However, activation of DR4 and DR5 seems to occur via intracellular, membrane bound TRAIL. Thereby, TRAIL, DR4 and DR5 are specified as rate-limiting factors for 5-FU-induced toxicity, and in agreement with previous reports, p53 contributes to transcriptional regulation of the corresponding genes [18, 38]. Delimitation of either factor may consequently be the explanation for why suboptimal apoptosis occurs in cells lacking the tumor suppressor. Since preclinical models demonstrated that recombinant TRAIL has potent anti-tumor activity without exhibiting systemic cytotoxicity [39], DR4 and DR5 were postulated as potential targets for future cancer therapeutics. In fact, agents with pronounced anti-tumorigenic potential, such as inhibitors of histone deacetylases (HDACIs), retinoids and interferons, depend on TRAIL expression and the subsequent activation of death receptors for the execution of tumor cell death [18, 38]. The conclusion from the present work is that 5-FU may also stimulate a similar death process. It must be noted that details regarding the TRAIL-DR4/5-interaction requires further exploration. *In silico* analysis of TRAIL using an algorithm for the prediction of transmembrane helices and their orientation (TMHMM; Center for Biological Sequence Analysis, Technical University of Denmark) indicated the ligand as a mainly cytosolic type 1 integral membrane protein. Accordingly, soluble TRAIL (sTRAIL) is generated within cells and then exported for subsequent receptor oligomerization. A processed TRAIL variant was indeed detected in treated samples by SDS-PAGE, but loss of the band in 5-FU and zVAD-fmk co-treated cells suggests that the appearance of this potential sTRAIL occurs as a consequence of caspase activity. Since reduced but still significant induction of *DR4* and *DR5* gene transcription could also be detected in the absence of p53, it is clear that transcription factors separated from the p53 protein network support gene expression. For 5-FU-induced apoptosis, these factors remain elusive but zerumbone and celecoxib, two ER stress-inducing drugs, were recently shown to activate DR5 transcription by means of the activating transcription factor 4 (ATF4)/C/EBP homologous protein and CHOP/ATF3 transcriptional cascade in a p53-independent manner [19]. Whether p53-transcriptional regulation of Bax or PUMA also contributes to the efficiency of 5-FU-induced apoptosis has not been investigated. However, considering their central role in the deregulation of mitochondrial membrane potential, it is likely. Our data verify a signaling link between RNA-related stress and DISC-mediated apoptosis. Although RNA lesions seem to serve as a foundation for caspase cascade initiation, we cannot exclude the possibility that silent genomic stress is introduced by 5-FU metabolites, for example through FdUTP misincorporation. In fact, data revealing specific augmentation of caspase activation by olaparib and 5-FU co-treatment in p53-deficient cells is in support of such a scenario. Since PARP-1 has been implicated in the BER process, stalled enzyme efficiency induced by RNAi or chemical inhibitors may lead to a more prominent damaging effect of 5-FU on DNA [23]. Present data only allows us to speculate regarding the reasons why PARP-1 inhibition or RNAi silencing in combination with 5-FU primarily affects p53-negative HCT116 cells. One explanation could be synthetic lethality, where the absence of p53 renders cells with additional DNA repair defects and thus greater sensitivity to combined treatment with PARP-inhibitors and 5-FU. For example, it was shown that HR deficiency caused by phosphatase and tensin homolog (PTEN) mutations sensitizes tumor cells to PARP inhibitors, both *in vitro* and *in vivo* [40]. Similarly, selective anti-proliferative effects of BRCA1 or BRCA2 (breast cancer, early onset)-deficient tumors have been demonstrated with olaparib [41]. Alternatively, p53 has also been reported to stimulate BER directly [42], thus suggesting those cells lacking the tumor suppressor to be more susceptible to PARP inhibition. In conclusion, our results show that 5-FU can stimulate TRAIL- and DR4/5-dependent apoptosis, which is facilitated by p53 but still proceeds in the absence of the tumor suppressor. Although the dominant origin for the process seems to be RNA-related, further analyses should be designed to elucidate whether or not other stress targets of the drug also contribute to the chemotherapeutic response.

## MATERIALS AND METHODS

### Cell culture

The HCT116 parental cell line, its variants (kindly provided by Professor Bert Vogelstein) and RKO cells (ATCC) were cultured in Dulbecco's modified Eagle's medium (DMEM), supplemented with heat-inactivated FBS (10%) (GIBCO, Invitrogen, San Diego, CA, USA) and PenStrep (100 U/ml penicillin, 100 mg/ml streptomycin; Sigma-Aldrich, Saint Luis, MO, USA). Cell-treatment with the antimetabolite 5-FU (Accord Healthcare Ltd, Harrow, UK) was performed at concentrations and incubation-times as indicated in the figures. In selected experiments the synthetic pan-caspase inhibitor N-benzyloxycarbonyl-Val-Ala-Asp(O-Me) fluoromethyl ketone (zVAD-fmk, 10 μM) (Peptide Institute, Inc., Osaka, Japan), the PARP inhibitor olaparib (500 nM; Selleck Chemicals, Houston, TX, USA), E64D (10 μM; Sigma-Aldrich), pepstatin A (7 μM; Sigma-Aldrich), leupeptin (100 μM; Sigma-Aldrich), BAPTA (10 μM; Life Technologies, Carlsbad, CA, USA), recombinant soluble DR5 (2 μg/ml; Sigma-Aldrich), recombinant TRAIL (10 ng/ml; Life Technologies), leucovorin (Hospira Nordic AB, Stockholm, Sweden) or CA074 (10 μM; Sigma-Aldrich) were added to cell cultures 1 h prior to drug treatment. In addition, treatments of cells using doxorubicin (2 μM; Sigma-Aldrich), uridine (375 μM; Sigma-Aldrich), thymidine (375 μM; Sigma-Aldrich), etoposide (20 μM; Ebewe, Austria), cisplatin (40 μM; Ebewe, Austria) and camptothecin (600 nM; Sigma-Aldrich) were performed in selected experiments.

### Mitochondrial AIF and cytochrome c release

Detection of mitochondrial AIF and cytochrome *c* release was accomplished by the digitonin (Calbiochem, San Diego, CA USA) organelle fractionation procedure performed as described [43].

### Analysis of mitochondrial membrane potential and ROS

Mitochondrial membrane potential (ΔΨ_m_) was estimated with TMRE (tetra-methyl-rhodamine-ethyl ester, Life Technologies). Cells were incubated for 20 min in Hank's balanced salt solution (Life Technologies) containing 0.1 μM TMRE. For assessment of mitochondrial ROS, cells were stained with MitoSOX™ Red (Life Technologies) according to the manufacturer's recommendations. Analyses were accomplished by using a FACScan Becton Dickinson flow cytometer using the accompanying software.

### Gel electrophoresis and immunoblotting

SDS-PAGE was performed according to previously described procedures [6].

### DR5 dimerization assay

DR5 dimers were analyzed by SDS-PAGE using lysates isolated without reducing agent. Cells were treated with 5-FU, washed with PBS, and lysed for 20 min on ice in 20 mM Tris-HCl (pH 7.5), 150 mM NaCl, 10% glycerol, and 1% Triton X-100 supplemented with cOmplete phosphatase/protease inhibitors (Roche Diagnostics). After centrifuged at 16,000 × g at 4°C, the cleared lysates were divided into two aliquots and diluted with 3 × SDS sample buffer with or without β-mercaptoethanol. The samples containing reducing agent were incubated at 95°C for 5 min, whereas the non-reduced samples were kept at RT before loading.

### Expression vectors and retroviral transduction

The retroviral expression vectors pLXINhFADD and pXINhFLIP_L_ have been described previously [44]. Retroviral particles were produced by transient transfection of the Phoenix-Ampho packaging cell line (kindly provided by Dr. GP Nolan, Stanford University, USA). Production of viral particles and retroviral transductions were performed as described [44]. Transduced cells were selected by treatment with 0.8 mg/ml of Geneticin (Invitrogen).

### RNA isolation and RT-PCR

Total RNA was isolated from control and 5-FU-treated HCT116 *wt* and *p53^−/−^* cells with RNAeasy mini kit (Qiagen) according to the manufacturer's instructions. The Maxima First Strand cDNA Synthesis Kit was used for cDNA synthesis (Life Technologies). Gene expression was measured using Maxima SYBR Green qPCR Master Mix (Fermentas) with Applied Biosystems 7500 Real-time PCR (Applied Biosystems). Relative gene expression was calculated with 2^−ΔΔ^Ct method using Actin expression for normalization.

### Primers used

DR5 (F-TCAGGTGAAGTGGAGCTAAGTC, R-GTGTACAATCACCGACCTT),

DR4 (F-ACTCGCTGTCCACTTTCGTCTCTGA, R-AGGCATCCCCTGGGCCTGCTGTA), actin (F-GCTGTGCTATCCCTGTACGC, R-GAGGGCATACCCCTCGTAGA).

### Transmission electron microscopy

Cells were fixed in 2.5% (w/v) glutaraldehyde in 0.1 M phosphate buffer, pH 7.4 for 30 min at RT, and fixed in the same fixative in the refrigerator. After rinsing in 0.1 M phosphate buffer cells were centrifuged and the pellets post fixed in 2% (w/v) osmium tetroxide in 0.1 M phosphate buffer (pH 7.4) at 4°C for 2 h, dehydrated in ethanol followed by acetone, and embedded in LX-112 (Ladd). Ultrathin sections (∼40–50 nm) were cut using a Leica EM UC 6 ultramicrotome. Sections were subsequently contrasted with uranyl acetate followed by lead citrate and examined in a Tecnai 12 Spirit Bio TWIN transmission electron microscope (FEI) at 100 kV. Digital images were taken using a Veleta camera (Olympus Soft Imaging Solutions).

### Immunofluorescence

Immunocytochemistry was performed according to previously described procedures [6].

### Antibodies

The following primary antibodies were used in western blotting: anti-p53 monoclonal antibody, anti-AIF (Santa Cruz Biotechnology, Santa Cruz, CA, USA), anti-PAR mAb, anti-GAPDH polyclonal antibody (pAb; Trevigen, Gaithersburg, MD, USA), anti-phospho-p53 pAbs (Ser 15, 33, and 46), anti-cleaved-caspase-3 pAb, anti-γH2AX (Ser 139) pAb; cleaved PARP mAb (Asp214), cleaved lamin A mAb, PERK mAb (Cell Signaling, Danvers, MA, USA), anti-tubulin mAb, anti-DR5 pAb; anti-DR4 pAb (Sigma-Aldrich), anti-PARP mAb, anti-cytochrome *c*, anti-TRAIL mAb, Ku80 mAb (BD Biosciences, Franklin Lakes, NJ, USA), anti-cFLIP mAb (Alexis, San Diego, CA, USA) and anti-caspase-8 mAb (kindly provided by Professor PH Krammer and Dr. I Lavrik, German Cancer Research Center, Heidelberg, Germany). Analysis of DR5 in immunofluorescence was performed using the mAb F2/B4 (kindly provided by Dr. L Andêra, Academy of Sciences, Prague, Czech Republic). For other immunofluorescence stainings, antibodies identical to those in western blotting procedures were used. Fluorescent secondary antibodies directed against mouse and rabbit (Alexa488 and Alexa594) were purchased from Molecular Probes (Invitrogen).

### Measurement of caspase-3/-7-like activities

Measurement of the caspase-3/-7-like substrate Ac-DEVD-AMC (acetyl Asp-Glu-Val-Asp 7-amido- 4-methylcoumarin; Peptide Institute, Osaka, Japan) cleavage was performed as described [45].

### RNA interference

Transfection of TRAIL (siTNFSF10, s16665), DR5 (siTNFSF10B, s16756), DR4 (siTNFSF10A, s16764), negative control (siNegative Control No1) (silencer select, Life Technologies), p53 (siTP53-9, FlexiTube), PARP (siPARP1 HP Custom siRNA Qiagen, Hilden, Germany), caspase-8 (L-003466-00) and negative control (D-00120613) ONTARGET-plus SMARTpool siRNAs (Dharmacon, CO, Lafayette, USA) was performed using the Lipofectamine RNAiMAX transfection reagent (Life Technologies) according to the manufacturer's instructions.

### Colony assay

Eighteen cells per cm^2^ were seeded one day in advance of experimental onset. 5-FU at concentrations indicated in the figures was maintained for 48 h. Cell colonies were then allowed to form over 10 days before staining in a 0.04% (w/v) crystal violet solution.

### Lactate dehydrogenase (LDH) measurement

Analysis of homogeneous plasma membrane integrity was used as an indicator of necrosis by taking advantage of the fluorometric CytoTox-ONE™ Assay (Promega, Madison, WI, USA) and by following the manufacturer's recommendations.

### Cell cycle and sub G1 analysis

Harvested cells were fixed in 70% ethanol for 1 h at 4°C. Repeated washes in PBS and RNase A (100 μg/ml, Invitrogen) treatment for 1 h at 37°C were followed by propidium iodide staining (50 μg/ml, Sigma-Aldrich). Analysis in FL3 on DDM mode was performed using the BD Accuri C6 system in combination with BD CSampler software (Becton-Dickinson, San Jose, CA, USA).

### Immunoprecipitation of DR5

Immunoprecipitation of DR5 was performed using the protocol described and taking advantage of the specific DR5 mAb (clone F2/B4, 3 μg/ml IP buffer) [43].

### Statistical analysis

Results are expressed as the mean ± standard deviation (SD). GraphPad Prism 5.02 software and *t*-test were used in analysis.

## SUPPLEMENTARY FIGURES


