# Correction: 5-Fluorouracil-induced RNA stress engages a TRAIL-DISC-dependent apoptosis axis facilitated by p53

**DOI:** 10.18632/oncotarget.9248

**Published:** 2016-05-09

**Authors:** Birce Akpinar, Ethiene V. Bracht, Dorin Reijnders, Barbora Safarikova, Iva Jelinkova, Alf Grandien, Alena Hyrslova Vaculova, Boris Zhivotovsky, Magnus Olsson

Present: Due to a technical error during image processing, the same picture set of control cells were used in TEM figures for both HCT116 wt and p53−/− cells. Figure 2B and Supplementary Figure 2 were affected.

Corrected: Correct Figure 2B and Supplementary Figure 2 are provided below. Authors sincerely apologize for this oversight.

Original article: Oncotarget. 2015; 6(41): 43679-97. doi: 10.18632/oncotarget.6030.

**Figure 2B:**
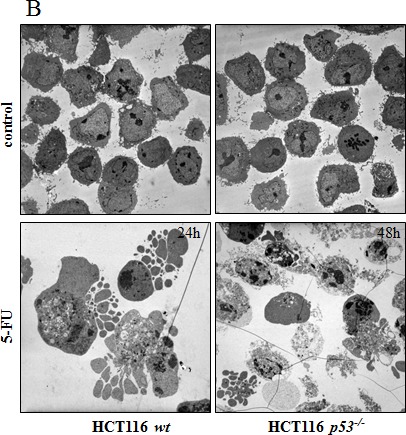
Analysis of LDH-release from HCT116 *p53^−/−^* cells at 24, 48 and 72 h post-5-FU treatment (768 μM), either in the presence or absence of the pan-caspase inhibitor zVAD-fmk (10 μM).

## SUPPLEMENTARY FIGURES



