# Correction: 5-Fluorouracil-induced RNA stress engages a TRAIL-DISC-dependent apoptosis axis facilitated by p53

**DOI:** 10.18632/oncotarget.12849

**Published:** 2016-10-13

**Authors:** Birce Akpinar, Ethiene V. Bracht, Dorin Reijnders, Barbora Safarikova, Iva Jelinkova, Alf Grandien, Alena Hyrslova Vaculova, Boris Zhivotovsky, Magnus Olsson

Present: Due to a processing mistake during production, the original correction notice issued for this article contained two errors. Specifically, it provided corrections to supplementary figure S2, but should have provided corrections to supplementary figure S3. It also provided incorrect figure captions for Figure 2B and Supplementary Figure S3.

Corrected: Corrected figure designations and captions are provided below. The publisher sincerely apologizes for this oversight.

Original article: Oncotarget. 2015; 6(41):43679-97. doi: 10.18632/oncotarget.6030.

First Correction: Oncotarget. 2016; 7(19):28761-62. doi: 10.18632/oncotarget.9248.

**Figure 2B:** Representative transmission electron microscope images of sections prepared from control and 5-FU-treated wtand *p53^−/−^* cells. An isolation of dead cells was performed in treated samples by collecting floating cells prior to fixation and section preparation.

**Supplementary Figure S3: Representative transmission electron microscope images of sections prepared from control and 5-FU-treated (36 h) HCT116 *p53^−/−^* cells.** An isolation of dead cells was performed in treated samples by collecting floating cells by means of centrifugation prior to fixation and section preparation.

