# Supplementary material for: 5-Fluorouracil-induced RNA stress engages a TRAIL-DISC-dependent apoptosis axis facilitated by p53
Source: Oncotarget. 2015 Oct 24;6(41):43679–97. doi: 10.18632/oncotarget.6030 (PMC4791259; doi:10.18632/oncotarget.6030)
Supplement: Supplementary file 1 [file oncotarget-06-43679-s001.pdf]

SUPPLEMENTARY FIGURES

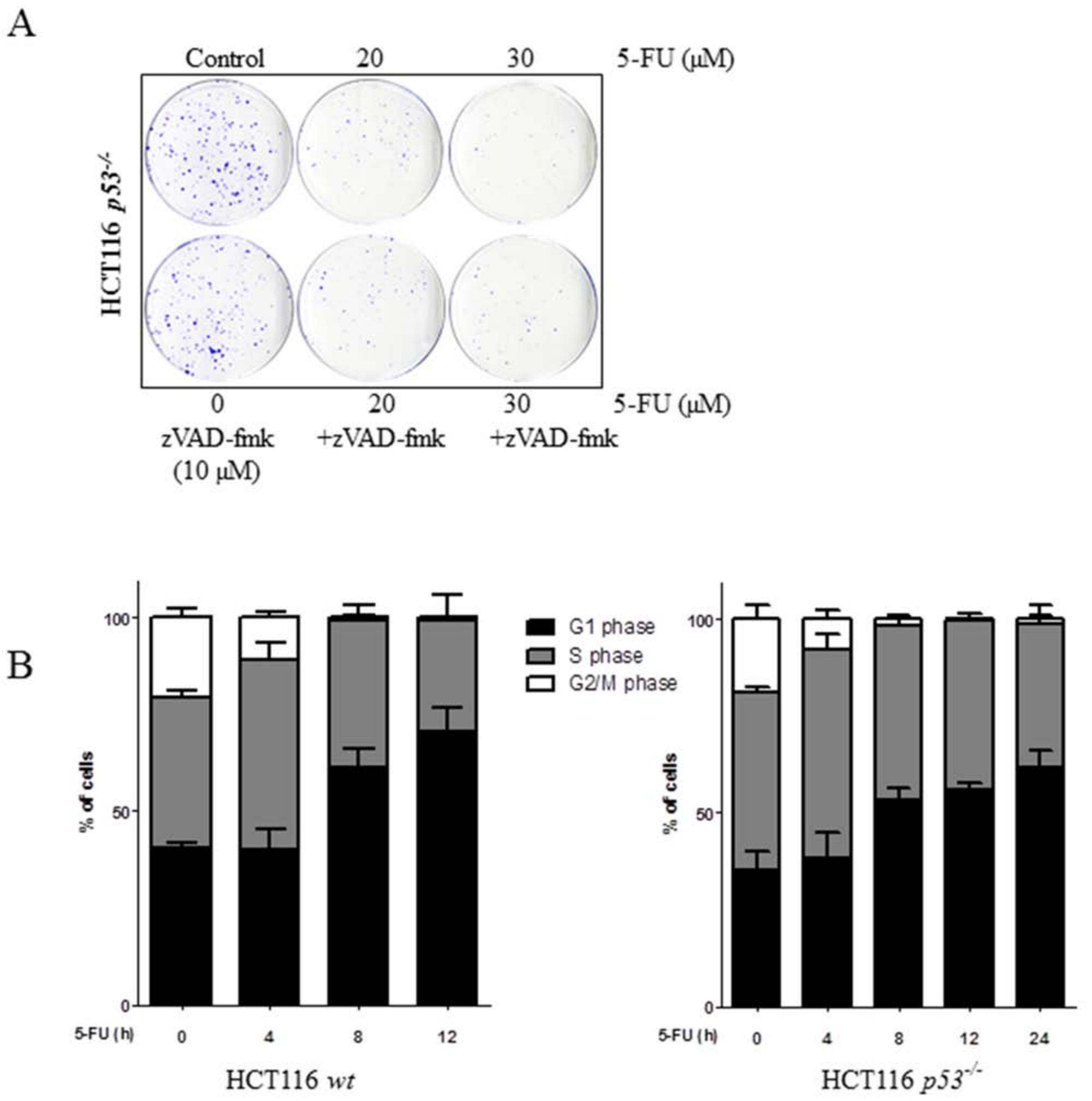

Supplementary Figure S1: Reduced colony forming capacity of 5-FU-treated HCT116 cells is a consequence of rapid and p53-independent cell cycle arrest. Clonal survival of eighteen HCT116 *p53*$^{-/-}$cells/cm^2 in response to 20 or 30 µM 5-FU were estimated, either in the presence or absence of the pan-caspase inhibitor zVAD-fmk (10 µM, 48 h) **A.** In **B.** cells were cultured up to 70% confluence before treatment with 5-FU (350 µM). After induction, cells were harvested and subjected to propidium iodide staining as described in materials and methods. Finally, FACS analysis was performed using the FACScan system and the Modfit software (BD, Bioscience). Statistical analysis was performed using a Bonferroni's Multiple Comparison test. Bars represent mean + SEM of minimal three experiments. No significant differences.

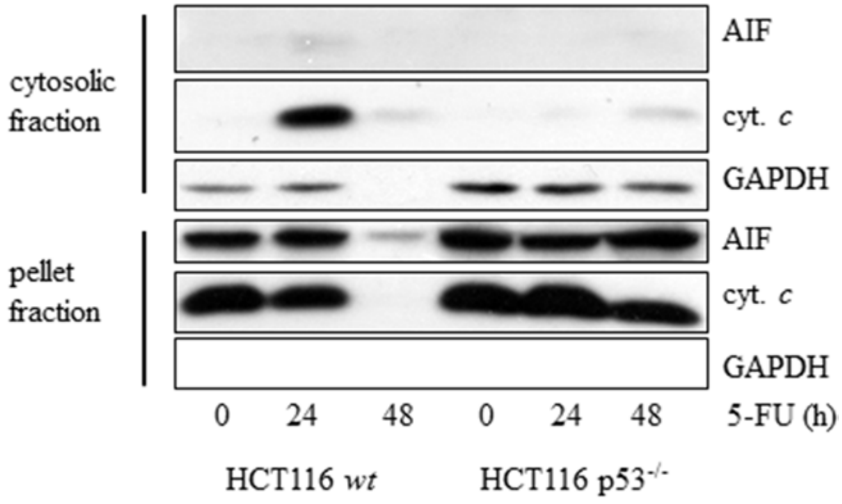

Supplementary Figure S2: Mitochondrial release of cytochrome c but not AIF in 5-FU-treated HCT116 wt and p53$^{-/-}$ cells. HCT116 *wt* and *p53$^{-/-}$* cells, treated for 24 or 48 h were, along with non-treated controls, fractionated into cytoplasmic and mitochondrial/nuclear protein pools. Samples were separated by SDS-PAGE and cytoplasmic presence of either AIF or cytochrome *c* analyzed by immunoblotting. GAPDH served both as a marker for equal sample loading and as an indicator of fractionation efficacy.

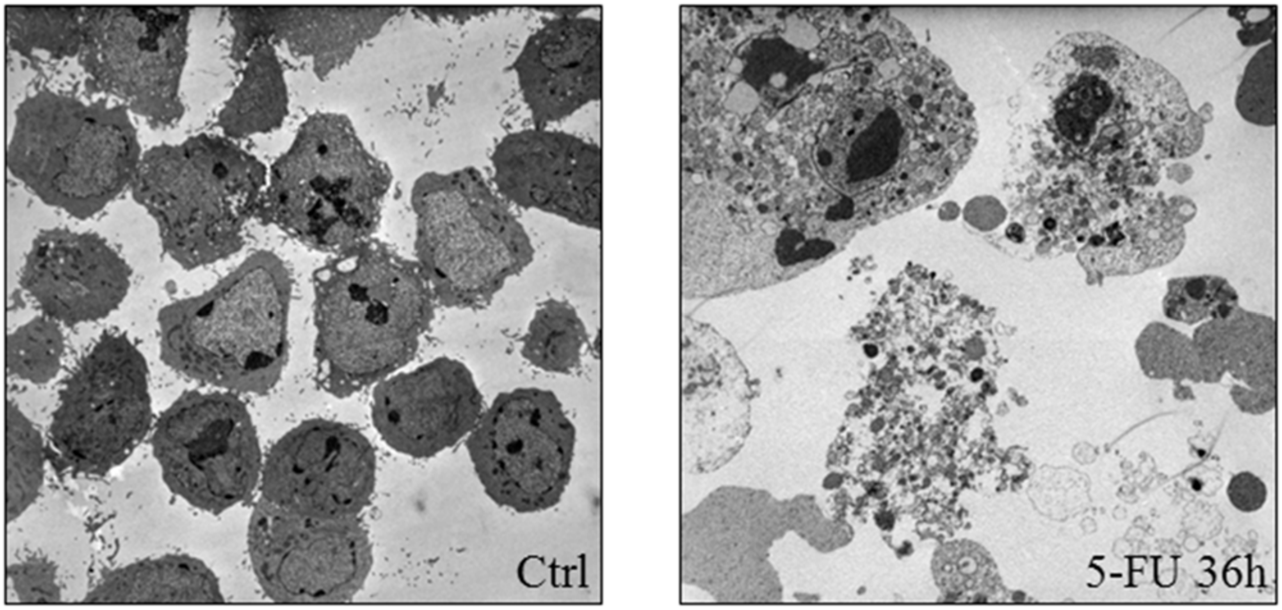

Supplementary Figure S3: Necrotic morphology of HCT116 p53$^{-/-}$ cells treated with 5-FU for 36 h. Representative transmission electron microscope images of sections prepared from control and 5-FU-treated (36 h) *HCT116 p53$^{-/-}$* cells. An isolation of dead cells was performed in treated samples by collecting floating cells by means of centrifugation prior to fixation and section preparation.

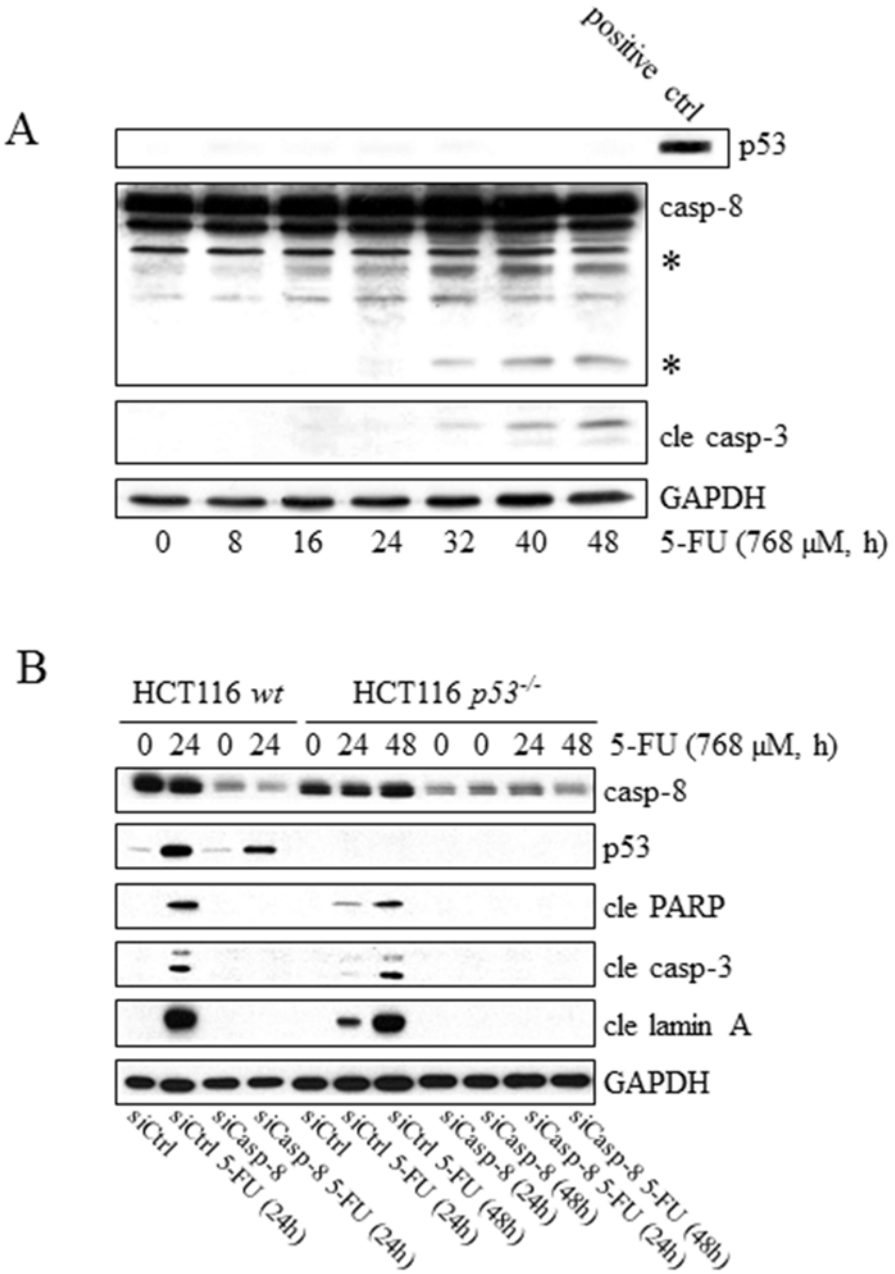

Supplementary Figure S4: Apical caspase-8 is required for efficient effector caspase-3 processing occurring as a result of 5-FU toxicity in HCT116 p53$^{-/-}$ cells. The effect of 5-FU with respect to the processing of apical caspase-8 and effector caspase-3 in HCT116 *p53*$^{-/-}$ cells was analyzed by immunoblotting of total protein lysates isolated at different time points following treatment. A protein lysate isolated from 5-FU-induced HCT116 *wt* cells was used as a positive control for the p53 antibody **A.** The importance of caspase-8 for apoptotic proceedings initiated by 5-FU was analyzed by RNAi. Using SDS PAGE, lysates from sicaspase-8 and si-control-transfected HCT116 *wt* and *p53*$^{-/-}$ cells were analyzed with respect to status of processed caspase-3, PARP and lamin A **B.** GAPDH served as a marker for equal sample loading. Processed caspase-8 fragments are indicated with asterisks (A).

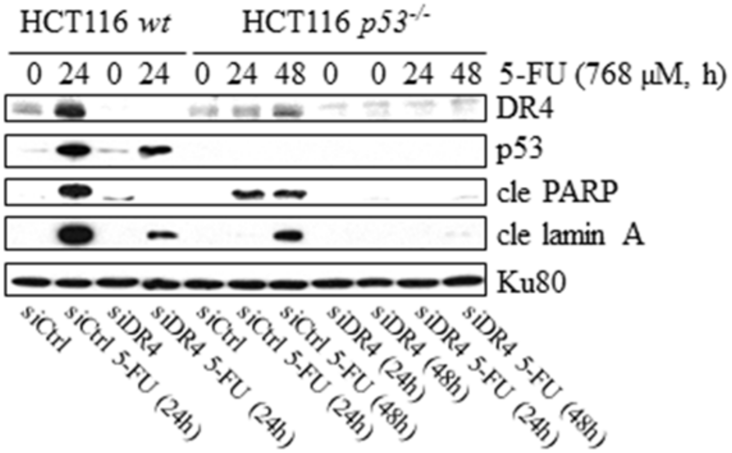

Supplementary Figure S5: DR4 is required for efficient apoptosis occurring in response to 5-FU treatment in HCT116 wt and p53$^{-/-}$ cells. Immunoblot-detection of apoptosis markers (cle PARP, cle lamin A) in lysates from 5-FU-treated HCT116 *wt* and *p53*$^{-/-}$ cells in which DR4 suppression was accomplished by means of RNAi. Comparisons of si control (siCtrl) and siDR4 transfected cells are outlined. Ku80 was used as a control for equal loading of samples.

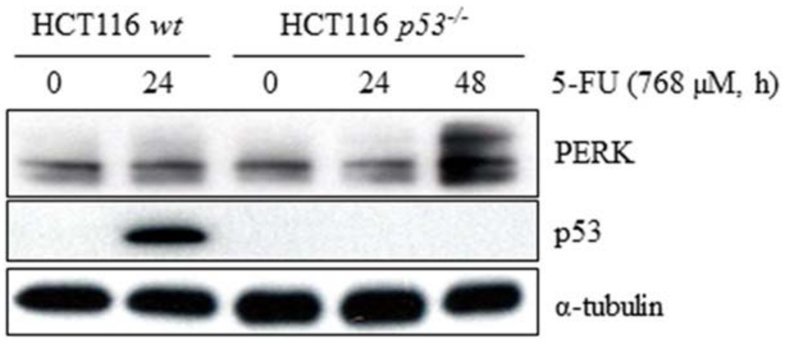

Supplementary Figure S6: In the absence of p53, 5-FU-treatment follows by ER-stress. Immunoblot-detection of the ER-stress marker PERK in lysates from 5-FU-treated HCT116 *wt* and *p53*$^{-/-}$ cells. α-tubulin was used as a control for equal loading of samples.

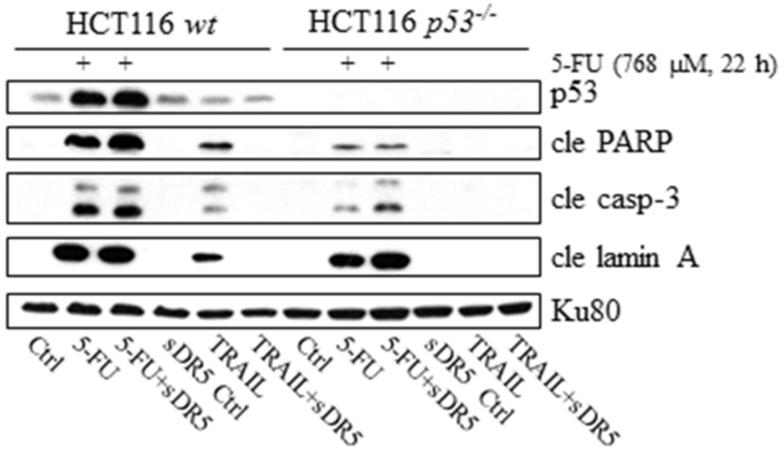

Supplementary Figure S7: Extracellular TRAIL is not required for 5-FU-induced apoptosis. HCT116 *p53*$^{-/-}$ and *wt* cells treated with 5-FU (768 µM, 22 h) or recombinant TRAIL (10 ng/ml), either alone or in combination with a soluble recombinant DR5 (2 µg/ml; sDR5) were harvested and their lysates subjected to SDS-PAGE. Electro-blotted nitrocellulose membranes were then probed with p53 and several apoptotic protein markers (cle PARP, cle casp-3 and cle lamin A). Ku80 was used as a control for equal loading of samples.

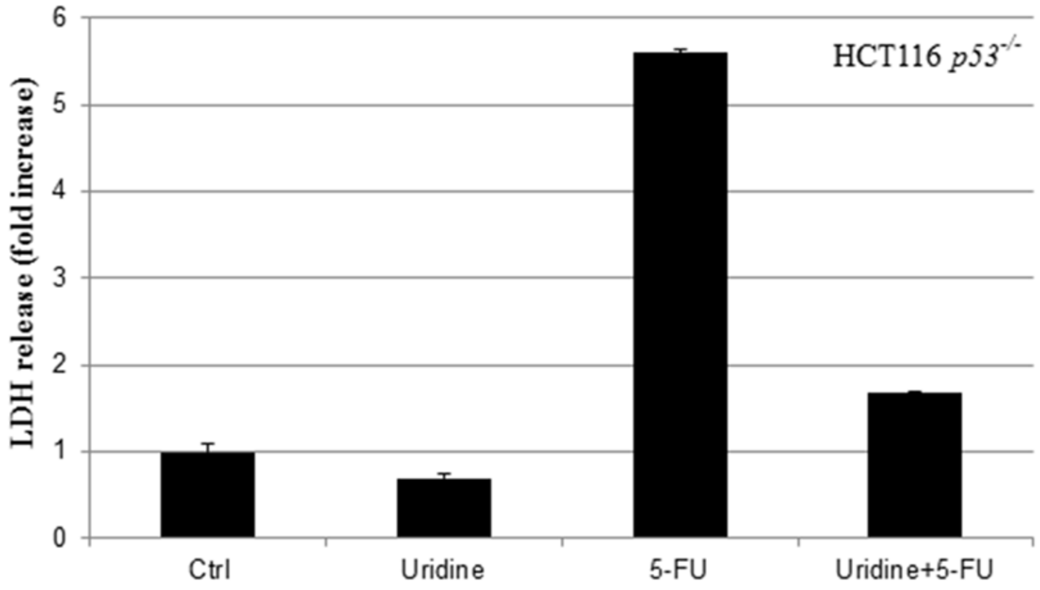

Supplementary Figure S8: Uridine inhibits 5-FU-induced LDH-release in HCT116 p53$^{-/-}$ cells. Analysis of LDH-release from HCT116 *p53*$^{-/-}$ cells at 48 h post-5-FU treatment (384 µM), either in the presence or absence of uridine (384 µM).

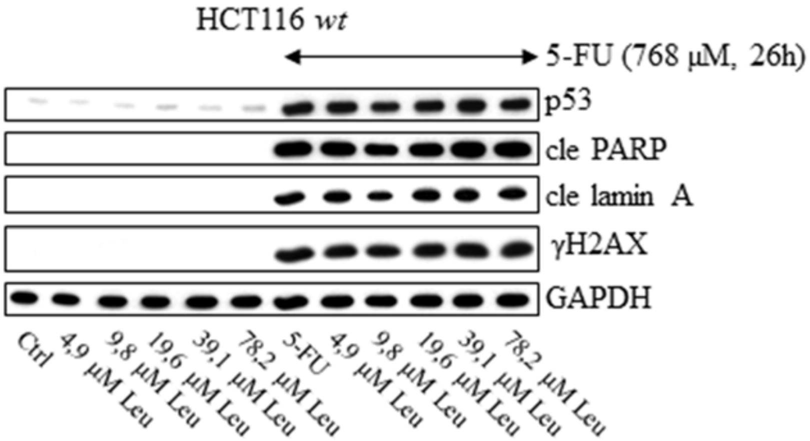

Supplementary Figure S9: Co-treatment using leucovorin do not reinforce 5-FU-induced apoptosis. HCT116 *wt* cells treated with leucovorin (leu) (concentrations ranging from 4.9 to 78.2 µM), either alone or in combination with 5-FU (768 µM, 26 h) were harvested and their lysates subjected to SDS-PAGE. Electro-blotted nitrocellulose membranes were then probed with p53, γH2AX and several apoptotic protein markers (cle PARP and cle lamin A). GAPDH was used as a control for equal loading of samples.

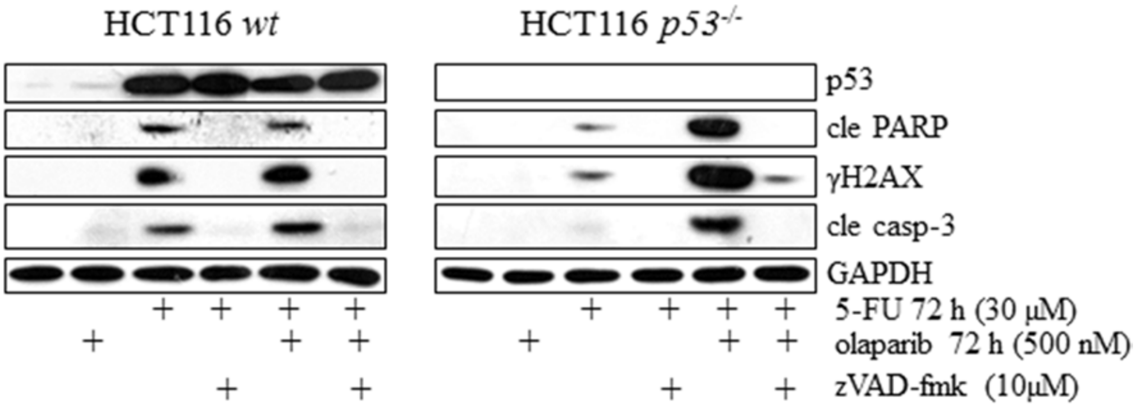

Supplementary Figure S10: Sensitization of tumor cell lines to 5-FU by olaparib occurs specifically in the absence of p53. The apoptotic effects of 5-FU (30 μM) and olaparib (500 nM), either alone or in co-treatments was analyzed by immunoblotting of HCT116 *p53*$^{-/-}$ and *wt* cell lysates. Electro-blotted nitrocellulose membranes were then probed with p53, γH2AX and apoptotic protein markers (cle PARP and cle casp-3). GAPDH was used as a control for equal loading of samples.
