# Supplementary material for: Correction: 5-Fluorouracil-induced RNA stress engages a TRAIL-DISC-dependent apoptosis axis facilitated by p53
Source: Oncotarget. 2016 May 9;7(19):28761. doi: 10.18632/oncotarget.9248 (PMC5053761; doi:10.18632/oncotarget.9248)
Supplement: Supplementary file 1 [file oncotarget-07-28761-s001.pdf]

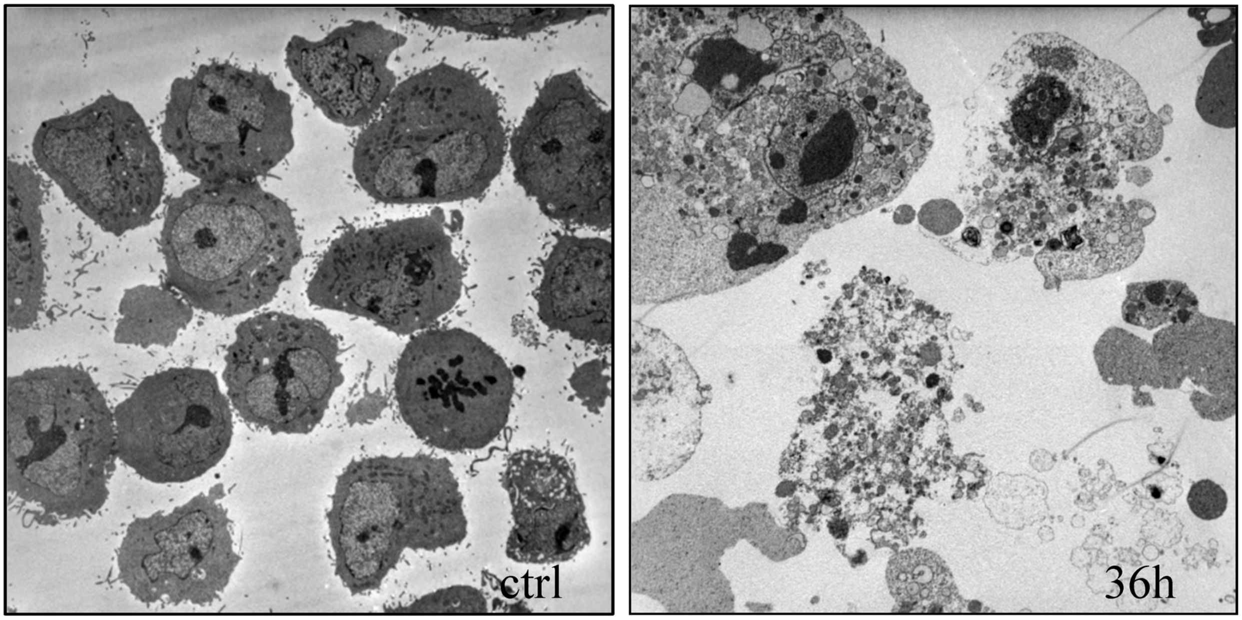

Supplementary Figure S2: Mitochondrial release of cytochrome c but not AIF in 5-FU-treated HCT116 wt and p53$^{-/-}$ cells. HCT116 *wt* and p53$^{-/-}$ cells, treated for 24 or 48 h were, along with non-treated controls, fractionated into cytoplasmic and mitochondrial/nuclear protein pools. Samples were separated by SDS-PAGE and cytoplasmic presence of either AIF or cytochrome *c* analyzed by immunoblotting. GAPDH served both as a marker for equal sample loading and as an indicator of fractionation efficacy.
